# Risk factors for extrapulmonary dissemination of tuberculosis and associated mortality during treatment for extrapulmonary tuberculosis

**DOI:** 10.1038/s41426-018-0106-1

**Published:** 2018-06-06

**Authors:** Xu Qian, Duc T. Nguyen, Jianxin Lyu, Andreas E. Albers, Xiaohong Bi, Edward A. Graviss

**Affiliations:** 1 Key Laboratory of Laboratory Medicine, Ministry of Education, Zhejiang Provincial Key Laboratory of Medical Genetics, Wenzhou Medical University, Wenzhou, P. R. China; 2 Center for Precision Biomedicine, Institute of Molecular Medicine, McGovern Medical School, The University of Texas Health Science Center at Houston, Houston, TX USA; 3 People’s Hospital of Hangzhou Medical College, Hangzhou, P. R. China; 4 Houston Methodist Research Institute, Houston, TX USA; 5 Department of Otorhinolaryngology, Head and Neck Surgery, Berlin Institute of Health, Charité – Universitätsmedizin Berlin, Corporate Member of Freie Universität Berlin, Humboldt-Universität zu Berlin, Campus Benjamin Franklin, Berlin, Germany

## Abstract

Many environmental, host, and microbial characteristics have been recognized as risk factors for dissemination of extrapulmonary tuberculosis (EPTB). However, there are few population-based studies investigating the association between the primary sites of tuberculosis (TB) infection and mortality during TB treatment. De-identified population-based surveillance data of confirmed TB patients reported from 2009 to 2015 in Texas, USA, were analyzed. Regression analyses were used to determine the risk factors for EPTB, as well as its subsite distribution and mortality. We analyzed 7007 patients with exclusively pulmonary TB, 1259 patients with exclusively EPTB, and 894 EPTB patients with reported concomitant pulmonary involvement. Age ≥45 years, female gender, human immunodeficiency virus (HIV)-positive status, and end-stage renal disease (ESRD) were associated with EPTB. ESRD was associated with the most clinical presentations of EPTB other than meningeal and genitourinary TB. Patients age ≥45 years had a disproportionately high rate of bone TB, while foreign-born patients had increased pleural TB and HIV+ patients had increased meningeal TB. Age ≥45 years, HIV+ status, excessive alcohol use within the past 12 months, ESRD, and abnormal chest radiographs were independent risk factors for EPTB mortality during TB treatment. The epidemiologic risk factors identified by multivariate analyses provide new information that may be useful to health professionals in managing patients with EPTB.

## Introduction

Tuberculosis (TB), especially with human immunodeficiency virus (HIV) co-infection, is a leading cause of death worldwide^1^. Individuals infected with *Mycobacterium tuberculosis* (*Mtb*) may either be asymptomatic (latent TB infection, LTBI) or develop active TB disease^2^. For active TB disease, a small subset of patients (19.3–39.3%) present with either primary extrapulmonary tuberculosis (EPTB) or EPTB concurrent with pulmonary involvement, while the majority of patients develop pulmonary TB (PTB)^3, 4^. Some studies have suggested that the proportion of EPTB among all TB cases has been increasing in the United States (USA) (21% in 2013 compared to 16% in 1993) mainly because of the increasing prevalence of HIV infection^5, 6^.

Typically, *Mtb* infection leads to spatial and temporal lesion dynamics not only within a single individual but also between individuals^7, 8^. The most common extrapulmonary sites of TB infection are the lymph nodes, the pleura, the genitourinary system, the gastrointestinal tract, the bones, and the central nervous system. To date, the mechanisms for extrapulmonary dissemination remain largely unknown^2^. It has been found that host–pathogen interactions such as pathogen-associated molecular pattern signaling, antigen presentation, and immune recognition may be used by *Mtb* to mediate latency induction and pathogen reactivation^2^. These factors are believed to be important in establishing the site of disease presentation and dissemination. One recent study, which assessed within-host bacterial population dynamics in a macaque TB model by using a genome barcoding system coupled with serial ^18^F-fluorodeoxyglucose radiotracers and positron emission tomography co-registered with computed tomography (PET/CT), suggested that in the first 6 weeks after infection, granuloma size but not bacterial burden is correlated with risk of local dissemination (<10 mm away) in the lungs^9^. Furthermore, genomic analyses of samples from lung and extrapulmonary biopsies of HIV-co-infected patients have demonstrated that the dissemination of *Mtb* from the lungs to extrapulmonary sites may occur as frequently as between lung sites^10^. Importantly, *Mtb* sub-lineages were differentially distributed throughout the lungs of these immunocompromised patients. Therefore, data from Lieberman and co-workers^10^ suggest that biopsies from the upper airway represent only a small fraction of the population diversity. These data are also consistent with a nonhuman-primate-model study which showed barcodes recovered from gastric and bronchoalveolar lavage samples represented only a fraction (3.75%) of all bacterial barcodes^9^. Additionally, there has been a study evaluating the immune response profile of inflammatory cytokines such as interferon-γ, interleukin (IL)-1β, and tumor necrosis factor (TNF)-β in HIV-negative children with TB disease^11^. At the time of TB diagnosis, the immune response in all pediatric TB patients (suppressed pro-inflammatory cytokines and increased regulatory T cell frequency) was not significantly different between PTB and EPTB patients. However, the recovery of the immune response was observed in children with PTB but not in children with EPTB after 6 months of TB treatment^11^. These findings suggest that the host immune response following treatment is specific to the disease (PTB vs. EPTB) rather than due to the within-host defense and cannot explain why one individual develops PTB while another develops EPTB.

Clinically, EPTB is still underrecognized, and diagnoses are often delayed due to its paucibacillary nature and atypical presentations. In fact, many characteristics such as HIV and female gender have been recognized as risk factors for EPTB dissemination^12–14^. However, there are few population-based studies in the USA investigating the association between primary sites of *Mtb* infection and mortality during TB treatment. For instance, one study analyzed the epidemiology and risk factors of EPTB from 1993 through 2006 but did not analyze risk factors for patient mortality^5^. Another study demonstrated risk factors for EPTB and mortality at 6 months after TB diagnosis from 1995 through 1999 in Harris Country, Texas, but this study analyzed data at the county level and not at the state or country level^15^. The third example is the association of *Mtb* lineage with the site of TB disease in a study that analyzed US data from 2004 through 2008; the study reported that the Euro-American, Indo-Oceanic, and East African-Indian bacterial lineages were found exclusively in EPTB^12^. Given the variety of organ-specific clinical scenarios and the nonspecific systemic symptoms of EPTB, a more profound understanding of the site distribution of EPTB, as well as the risk factors associated with extrapulmonary dissemination and mortality, is important for developing suitable protocols to manage EPTB patients. Accordingly, this analysis aimed to determine the characteristics associated with EPTB dissemination and mortality during TB treatment by using recent epidemiological data from Texas.

## Materials and methods

De-identified surveillance data of all confirmed TB patients reported to the Centers for Disease Control and Prevention’s TB Genotyping Information Management System (TBGMIS) between January 2009 and December 2015 from the state of Texas, USA, were analyzed. TB disease was classified as exclusively PTB, exclusively EPTB or EPTB with concurrent PTB involvement. Sites of EPTB include pleural, lymphatic, bone, genitourinary, peritoneal, and meningeal locations, among others. All patients received anti-TB treatment, and their outcomes were recorded as “completed”, “died”, or “unknown”.

Cases were categorized by site of disease. Differences across groups (exclusively PTB, exclusively EPTB and EPTB with concurrent PTB involvement) were determined by the chi-squared test or Fisher’s exact test as appropriate. Logistic regression was used to determine the characteristics that were associated with patients having exclusively PTB compared to individuals identified to have (1) exclusively EPTB, (2) EPTB with concurrent PTB involvement, or (3) any EPTB (patients with exclusively EPTB and EPTB with concurrent PTB involvement). Odds ratios (OR), adjusted odds ratios (^a^OR), and 95% confidence intervals (CI) were reported. Multiple logistic regression modeling was also used to determine the risk of patient mortality during treatment in patients with exclusively EPTB. Analyses were performed with SPSS 16.0 (SPSS, Inc., Chicago, Illinois, USA) and Stata MP14.2 (StataCorp LP, College Station, TX, USA). A *p*-value of <0.05 was considered statistically significant.

## Results

### Study population and characteristics

**Fig. 1:**
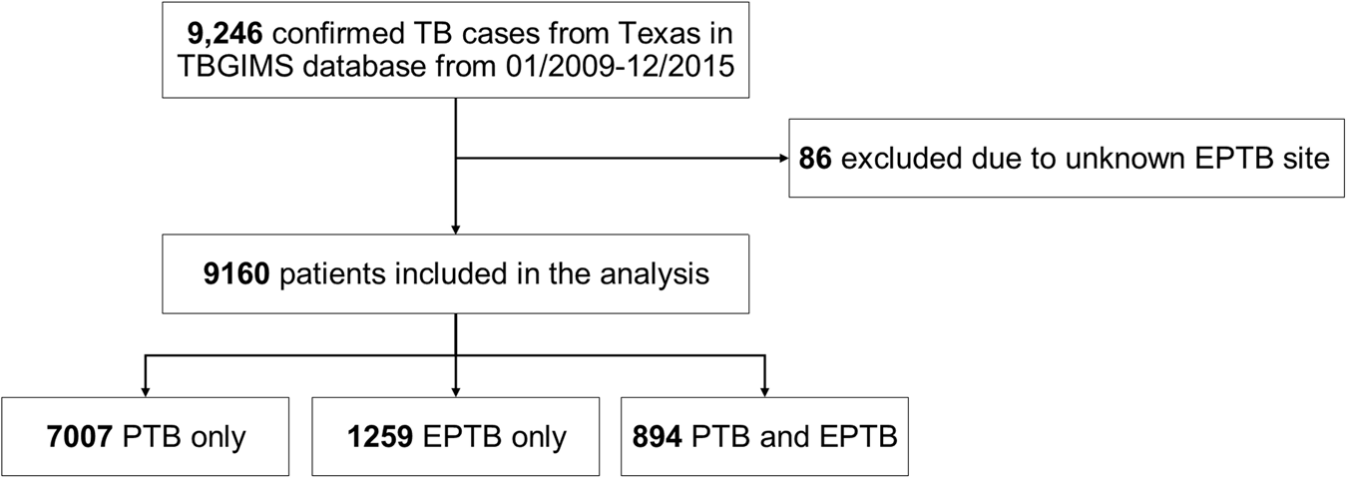
Flowchart of the study population. TBGIMS Genotyping Information Management System, PTB pulmonary TB, EPTB extrapulmonary TB

From 2009 to 2015, there were 9246 confirmed TB patients in Texas recorded in the TBGIMS database. After excluding 86 patients because their EPTB site was unknown, we included 9160 TB patients in the analysis (Fig. 1). The patients’ demographic and clinical characteristics are presented in Table 1. The majority of exclusively EPTB patients were male (55.4%) and foreign born (59.1%). The proportions of patients age 25–44 years (39.6%) and Hispanic patients (46.6%) with exclusively EPTB were higher than the proportions of other age or ethnic groups with exclusively EPTB. The percentage of TB contact history was higher in patients with exclusively EPTB (3.7%) than in patients with PTB (9.0%) or EPTB with concurrent PTB (6.9%). In patients with exclusively EPTB, 448/1259 (35.6%) had abnormal chest radiographs and 17/1259 (1.4%) had culture-positive specimens. Multidrug-resistant TB (MDR-TB) was identified in 0.4% of exclusively EPTB cases, 0.1% of EPTB cases with concurrent PTB, and 0.8% of exclusive PTB cases. Two extensively drug-resistant cases were identified in PTB patients. The most prevalent *Mtb* lineages of exclusively EPTB were Euro-American L4, East Asian L2, and Indo-Oceanic L1.

**Table 1:** Characteristics of tuberculosis patients with pulmonary and extrapulmonary locations in Texas, USA, 2009–2015

Variable	Total	Exclusively PTB	Exclusively EPTB	EPTB with PTB	*p*-Value
*N*	9160	7007	1259	894	
Age (years)					<0.01
0–4	395 (4.3)	297 (4.2)	63 (5.0)	35 (3.9)	
5–14	213 (2.3)	146 (2.1)	43 (3.4)	24 (2.7)	
15–24	1048 (11.4)	808 (11.5)	129 (10.2)	111 (12.4)	
25–44	3054 (33.3)	2245 (32.1)	498 (39.6)	311 (34.8)	
45–64	3015 (33.0)	2403 (34.3)	361 (28.7)	251 (28.1)	
≥65	1435 (15.7)	1108 (15.8)	165 (13.1)	162 (18.1)	
Gender					<0.01
Male	5954 (65.0)	4684 (66.8)	697 (55.4)	573 (64.1)	
Female	3206 (35.0)	2323 (33.2)	562 (45.6)	321 (35.9)	
Ethnicity					<0.01
White	1103 (12.0)	911 (13.0)	108 (8.6)	84 (9.4)	
Black	1717 (18.7)	1254 (17.9)	262 (20.8)	201 (22.5)	
Asian	1517 (16.6)	1052 (15.0)	297 (23.6)	168 (18.8)	
Hispanic	4771 (52.1)	3752 (53.6)	587 (46.6)	432 (48.3)	
Other	52 (0.6)	38 (0.5)	5 (0.4)	9 (1.0)	
HIV status					<0.01
Negative	7128 (77.8)	5518 (78.7)	959 (76.2)	651 (72.9)	
Positive	608 (6.7)	409 (5.9)	77 (6.1)	122 (13.6)	
Not offered	1424 (15.5)	1080 (15.4)	223 (17.7)	121 (13.5)	
Homeless					<0.01
No	8682 (94.8)	6600 (94.2)	1229 (97.6)	853 (95.4)	
Yes	478 (5.2)	407 (5.8)	30 (2.4)	41 (4.6)	
History of TB contact^a^					<0.01
No	8417 (91.9)	6373 (91.0)	1212 (96.3)	832 (93.1)	
Yes	743 (8.1)	634 (9.0)	47 (3.7)	62 (6.9)	
Excessive alcohol^b^					<0.01
No	7494 (81.8)	5633 (80.4)	1138 (90.4)	723 (80.9)	
Yes	1666 (18.2)	1374 (19.6)	121 (9.6)	171 (19.1)	
Injecting drug use					0.03
No	8930 (97.5)	6819 (97.3)	1241 (98.6)	870 (97.3)	
Yes	230 (2.5)	188 (2.7)	18 (1.4)	24 (2.7)	
Non-injecting drug use					
No	8263 (90.2)	6257 (89.3)	1202 (95.5)	804 (89.9)	<0.01
Yes	897 (9.8)	750 (10.7)	57 (4.5)	90 (10.1)	
Origin					0.01
US born	4108 (44.8)	3188 (45.5)	515 (40.9)	405 (45.3)	
Foreign born	5052 (55.2)	3819 (54.5)	744 (59.1)	489 (54.7)	
Diabetes					<0.01
No	7800 (85.2)	5883 (84.0)	1122 (89.1)	795 (88.9)	
Yes	1360 (14.8)	1124 (16.0)	137 (10.9)	99 (11.1)	
End-stage renal disease					<0.01
No	9024 (98.5)	6935 (99.0)	1230 (97.7)	859 (96.1)	
Yes	136 (1.5)	72 (1.0)	29 (2.3)	35 (3.9)	
Immunosuppression					0.01
No	8936 (97.6)	6854 (97.8)	1217 (96.7)	865 (96.8)	
Yes	224 (2.4)	153 (2.2)	42 (3.3)	29 (3.2)	
Previous TB					0.08
No	8819 (96.3)	6733 (96.1)	1226 (97.4)	860 (96.2)	
Yes	341 (3.7)	274 (3.9)	33 (2.6)	34 (3.8)	
Inmate of a correctional facility					<0.01
No	8210 (89.6)	6161 (87.9)	1192 (94.7)	857 (95.9)	
Yes	950 (10.4)	846 (2.1)	67 (5.3)	37 (4.1)	
Resident of long-term care facility					0.56
No	9046 (98.8)	6921 (98.8)	1240 (98.5)	885 (99.0)	
Yes	114 (1.2)	86 (1.2)	19 (1.5)	9 (1.0)	
Specimen smear					<0.01
Negative	4097 (44.7)	2832 (40.4)	707 (56.2)	558 (62.4)	
Positive	3585 (39.1)	3423 (48.9)	5 (0.4)	157 (17.6)	
Not done/Unknown	1478 (16.1)	752 (10.7)	547 (43.4)	179 (20.0)	
Specimen culture					<0.01
Negative	2449 (26.7)	1462 (20.9)	669 (53.1)	318 (35.6)	
Positive	5185 (56.6)	4782 (68.2)	17 (1.4)	386 (43.2)	
Not done/Unknown	1526 (16.7)	763 (10.9)	573 (45.5)	190 (21.3)	
Chest radiography					<0.01
Abnormal	7694 (84.0)	6485 (92.6)	448 (35.6)	761 (85.1)	
Normal	1024 (11.2)	233 (3.3)	700 (55.6)	91 (10.2)	
Not done/Unknown	442 (4.8)	289 (4.1)	111 (8.8)	42 (4.7)	
Radiographic cavity					<0.01
No	5140 (56.1)	4031 (57.5)	437 (34.7)	672 (75.2)	
Yes	2540 (27.7)	2441 (34.8)	11 (0.9)	88 (9.8)	
Unknown	1480 (16.2)	535 (7.7)	811 (64.4)	134 (15.0)	
DST profile					0.11
None to RIF/INH	6183 (67.5)	4902 (70.0)	684 (54.3)	597 (66.8)	
RIF or INH	512 (5.6)	420 (6.0)	44 (3.5)	48 (5.4)	
MDR	69 (0.7)	63 (0.8)	5 (0.4)	1 (0.1)	
XDR	2 (0.1)	2 (0.1)	0 (0.0)	0 (0.0)	
Unavailable	2394 (26.1)	1620 (23.1)	526 (41.8)	248 (27.7)	
Global *Mtb* Lineage					<0.01
Indo-Oceanic L1	634 (6.9)	483 (6.9)	93 (7.4)	58 (6.5)	
East Asian L2	1121 (12.2)	918 (13.1)	98 (7.8)	105 (11.7)	
East African-Indian L3	174 (1.9)	115 (1.6)	37 (2.9)	22 (2.5)	
Euro-American L4	4344 (47.4)	3569 (50.9)	376 (29.9)	399 (44.6)	
* M. bovis*	105 (1.1)	52 (0.8)	36 (2.9)	17 (1.9)	
Other	28 (0.3)	12 (0.2)	8 (0.6)	8 (0.9)	
Unknown	2754 (30.2)	1858 (26.5)	611 (48.5)	285 (31.9)	
Death at time of diagnosis					0.04
No	8964 (97.9)	6870 (98.0)	1220 (96.9)	874 (97.8)	
Yes	196 (2.1)	137 (2.0)	39 (3.1)	20 (2.2)	
Death during TB treatment					<0.01
No	8610 (94.0)	6592 (94.1)	1209 (96.0)	809 (90.5)	
Yes	551 (6.0)	416 (5.9)	50 (4.0)	85 (9.5)	

*PTB* pulmonary tuberculosis, *EPTB* extrapulmonary tuberculosis, *HIV* human immunodeficiency virus, *RIF* rifampin, *INH* isoniazid, *MDR* multidrug resistant, *XDR* extensively drug resistant. Differences across groups were compared using the chi-square test or Fisher’s exact test, as appropriate

^a^Patients with a history of contact with a known TB index case within 2 years

^b^Excessive alcohol use within the past 12 months

### Sites of EPTB

**Fig. 2:**
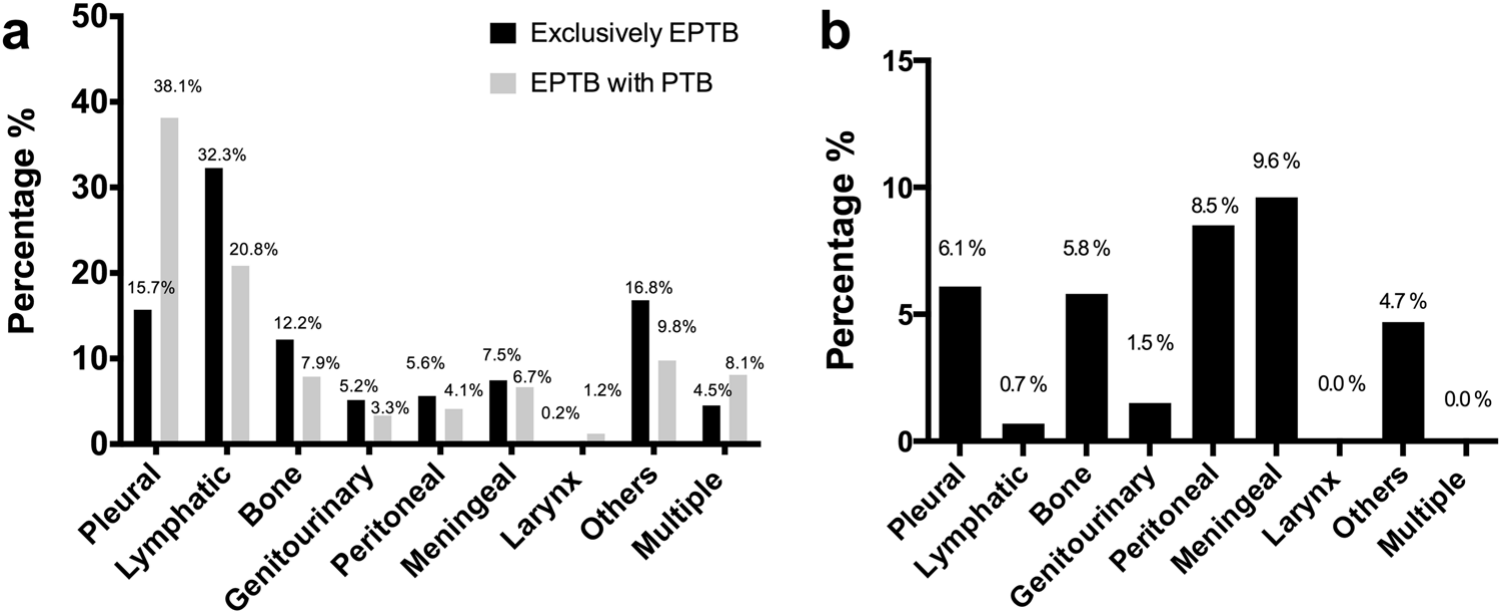
Distribution of extrapulmonary tuberculosis by site and associated mortality. **a** Distribution of extrapulmonary sites in patients with concurrent pulmonary tuberculosis (PTB) or exclusively extrapulmonary tuberculosis (EPTB). **b** Mortality distribution by extrapulmonary sites in patients with exclusively EPTB

The distribution of EPTB sites is shown in Fig. 2a. Of the patients with exclusively EPTB, the most common sites of TB disease included pleural (15.7%), lymphatic (32.3%), bone (12.2%), and meningeal (7.5%) sites. The most common sites of TB disease in patients having EPTB with concomitant PTB were also pleural (38.1%), lymphatic (20.8%), bone (7.9%), and meningeal (6.7%) areas.

### Risk factors for EPTB and its specific sites

Multivariable analyses were performed in order to identify associations between sociodemographic, microbiologic, and clinical characteristics of EPTB patients and sites of EPTB. Female patients (^a^OR 1.32, 95% CI 1.19–1.46), as well as patients with HIV+ status (^a^OR 1.77, 95% CI 1.47–2.13), immunosuppression (^a^OR 1.31, 95% CI 1.00–1.77), and ESRD (^a^OR 3.42, 95% CI 2.39–4.88) were at a significantly elevated risk of EPTB (Table 2). Patients with a history of contact with a known TB index case within 2 years (^a^OR 0.44, 95% CI 0.35–0.55) and those with diabetes (^a^OR 0.61, 95% CI 0.52–0.71) were less likely to have EPTB than PTB (Table 2).

**Table 2:** Multivariable analyses of patients’ characteristics with extrapulmonary tuberculosis in Texas, USA, 2009–2015

Characteristics	Exclusively EPTB vs. exclusively PTB		EPTB with PTB vs. exclusively PTB		Any EPTB vs. exclusively PTB	
	OR (95% CI)	^a^OR (95% CI)	OR (95% CI)	^a^OR (95% CI)	OR (95% CI)	^a^OR (95% CI)
Age (years)						
0–4	(reference)	(reference)	(reference)	(reference)	(reference)	(reference)
5–14	1.42 (1.04–1.96)	1.19 (0.85–1.68)	0.81 (0.55–1.19)	0.96 (0.62–1.49)	1.12 (0.86–1.50)	0.97 (0.74–1.28)
15–24	**1.98 (1.356**–**2.88)**	**2.33 (1.56**–**3.46)**	1.12 (0.71–1.79)	1.23 (0.76–1.99)	1.56 (1.14–2.13)	1.33 (0.96–1.84)
25–44	1.07 (0.837–1.37)	1.10 (0.85–1.42)	0.90 (0.73–1.22)	0.93 (0.71–1.21)	1.01 (0.83–1.22)	0.97 (0.80–1.18)
45–64	**1.49 (1.232**–**1.80)**	**1.57 (1.29**–**1.91)**	0.95 (0.77–1.16)	0.80 (0.67–1.03)	1.22 (1.05–1.42)	1.13 (0.72–1.32)
≥65	1.01 (0.828–1.23)	1.22 (0.99–1.49)	**0.71 (0.58–0.88)**	**0.67 (0.54–0.83)**	0.86 (0.74–1.01)	1.06 (0.91–1.24)
Gender (female)	**1.36 (1.20**–**1.54)**	**1.42 (1.26**–**1.62)**	1.12 (0.96–1.30)	1.16 (1.00–1.36)	**1.41 (1.27**–**1.55)**	**1.32 (1.19**–**1.46)**
Ethnicity						
White	(reference)	(reference)	(reference)	(reference)	(reference)	(reference)
Black	0.84 (0.33–2.15)	0.92 (0.35–2.38)	**0.39 (0.18–0.83)**	**0.37 (0.17–0.80)**	0.57 (0.30–1.08)	0.53 (0.28–1.01)
Asian	**0.76 (0.61–0.94)**	0.80 (0.63–1.02)	0.68 (0.32–1.42)	0.57 (0.17–1.20)	1.00 (0.54–1.87)	0.87 (0.46–1.63)
Hispanic	**1.34 (1.14**–**1.57)**	**1.34 (1.12**–**1.60)**	0.67 (0.32–1.42)	0.67 (0.31–1.45)	1.20 (0.64–2.24)	1.03 (0.54–1.95)
Other	**1.81 (1.55**–**2.11)**	**1.57 (1.34**–**1.85)**	0.49 (0.23–1.01)	0.48 (0.23–1.02)	0.74 (0.40–1.37)	0.68 (0.37–1.28)
Homeless	**0.56 (0.38–0.83)**	**0.57 (0.40–0.84)**	0.78 (0.56–1.08)	0.70 (0.50–1.00)	**0.55 (0.43–0.72)**	**0.65 (0.49–0.85)**
History of TB contact^a^	**0.39 (0.29–0.53)**	**0.28 (0.21–0.39)**	**0.75 (0.57–0.98)**	**0.71 (0.53–0.96)**	**0.54 (0.44–0.66)**	**0.44 (0.35–0.55)**
Excessive alcohol^b^	**0.61 (0.49–0.75)**	**0.60 (0.48–0.74)**	1.12 (0.91–1.38)	1.03 (0.84–1.26)	**0.64 (0.56–0.74)**	**0.78 (0.67–0.91)**
Injecting drug use	1.08 (0.64–1.82)	1.04 (0.62–1.76)	1.00 (0.63–1.60)	0.96 (0.60–1.53)	0.72 (0.52–1.01)	1.01 (0.70–1.46)
Non-injecting drug use	**0.53 (0.39–0.72)**	**0.52 (0.40–0.71)**	0.87 (0.67–1.14)	0.85 (0.65–1.11)	**0.61 (0.51–0.74)**	**0.68 (0.55–0.84)**
Diabetes	**0.63 (0.51–0.77)**	**0.61 (0.50–0.74)**	**0.65 (0.51–0.82)**	**0.61 (0.49–0.77)**	**0.64 (0.56–0.75)**	**0.61 (0.52–0.71)**
HIV^c^	1.14 (0.87–1.49)	1.20 (0.92–1.55)	**2.65 (2.10**–**3.35)**	**2.57 (2.05**–**3.21)**	**1.64 (1.38**–**1.96)**	**1.77 (1.47**–**2.13)**
Immunosuppression	1.44 (0.10–2.07)	1.36 (0.95–1.95)	1.20 (0.78–1.83)	1.15 (0.75–1.76)	**1.53 (1.15**–**2.03)**	**1.31 (1.00**–**1.77)**
ESRD	**2.51 (1.58**–**3.99)**	**2.59 (1.63**–**4.17)**	**4.30 (2.78**–**6.64)**	**4.45 (2.88**–**6.86)**	**3.32 (2.33**–**4.75)**	**3.42 (2.39**–**4.88)**
Foreign born	1.04 (0.89–1.22)	0.92 (0.80–1.06)	1.06 (0.89–1.28)	0.99 (0.85–1.17)	1.05 (0.93–1.20)	0.95 (0.85–1.06)
Previous TB	0.68 (0.47–1.00)	0.69 (0.47–1.01)	0.99 (0.68–1.43)	0.95 (0.66–1.38)	0.81 (0.61–1.07)	0.80 (0.61–1.06)

*EPTB* extrapulmonary tuberculosis, *PTB* pulmonary tuberculosis, *OR* odds ratio, ^*a*^*OR* adjusted odds ratio, *CI* confidence interval, *HIV* human immunodeficiency virus, *ESRD* end-stage renal disease, *TB*, tuberculosis

^a^Patients with a history of contact with a known TB index case within 2 years

^b^Excessive alcohol use within the past 12 months

^c^HIV unknown categorized as negative

Significant odds ratios (p<0.005) are in bold

ESRD was associated with most subtypes of EPTB, excluding meningeal and genitourinary TB (Table 3). Patients age ≥45 years had a disproportionately high rate of bone TB (^a^OR 1.47, 95% CI 1.04–2.08), while foreign-born patients had more pleural TB (^a^OR 1.77, 95% CI 1.31–2.41) and HIV+ patients had more meningeal TB (^a^OR 5.73, 95% CI 3.43–9.56) (Table 3).

**Table 3:** Risk factors associated with subsites of exclusively extrapulmonary tuberculosis compared to pulmonary tuberculosis in Texas, USA, 2009–2015

Characteristics	Pleural (*n* = 198)	Lymphatic (*n* = 407)	Bone (*n* = 154)	Genitourinary (*n* = 65)	Peritoneal (*n* = 71)	Meningeal (*n* = 94)	Others (*n* = 212)
	^a^OR (95% CI)	^a^OR (95% CI)	^a^OR (95% CI)	^a^OR (95% CI)	^a^OR (95% CI)	^a^OR (95% CI)	^a^OR (95% CI)
Age (≥45 years)	1.04 (0.76–1.40)	0.56 (0.44–0.71)	**1.47 (1.04**–**2.08)**	1.00 (0.60–1.68)	1.06 (0.64–1.74)	0.75 (0.48–1.17)	1.06 (0.79–1.42)
Gender (female)	0.91 (0.67–1.25)	**2.04 (1.66**–**2.51)**	**0.69 (0.48–0.99)**	1.44 (0.87–2.40)	**1.96 (1.20**–**3.21)**	1.22 (0.79–1.88)	**1.39 (1.05**–**1.86)**
Ethnicity (White)	0.73 (0.46–1.14)	0.47 (0.29–0.77)	1.14 (0.69–1.88)	1.26 (0.50–3.22)	0.62 (0.23–1.63)	0.82 (0.39–1.72)	0.77 (0.45–1.30)
Homeless	0.90 (0.48–1.68)	**0.29 (0.09–0.92)**	0.54 (0.19–1.50)	0.47 (0.06–3.57)	0.69 (0.16–2.95)	0.38 (0.09–1.63)	0.72 (0.29–1.82)
Excessive alcohol^a^	1.17 (0.80–1.71)	**0.38 (0.23–0.63)**	**0.42 (0.23–0.75)**	0.82 (0.35–1.90)	1.50 (0.77–2.95)	0.54 (0.26–1.14)	**0.30 (0.16–0.57)**
Injecting drug use	0.91 (0.35–2.34)	0.63 (0.15–2.68)	0.87 (0.20–3.79)	**5.26 (1.10**–**25.36)**	0.80 (0.10–6.36)	2.20 (0.60–8.05)	0.84 (0.20–3.62)
Non-injecting drug use	0.82 (0.49–1.38)	**0.37 (0.18–0.74)**	0.70 (0.33–1.53)	0.12 (0.02–1.05)	0.58 (0.19–1.76)	0.39 (0.14–1.07)	0.58 (0.27–1.24)
Diabetes	0.68 (0.43–1.07)	**0.35 (0.23–0.54)**	0.92 (0.60–1.42)	0.54 (0.24–1.22)	0.71 (0.35–1.44)	0.51 (0.23–1.13)	0.84 (0.56–1.26)
HIV^b^	0.62 (0.30–1.28)	1.20 (0.74–1.95)	0.54 (0.19–1.50)	0.64 (0.15–2.66)	1.13 (0.40–3.17)	**5.73 (3.43**–**9.56)**	1.52 (0.86–2.68)
Immunosuppression	0.80 (0.28–2.23)	0.88 (0.41–1.90)	**2.54 (1.30**–**4.97)**	1.57 (0.36–6.78)	2.29 (0.77–6.85)	1.79 (0.62–5.12)	0.95 (0.38–2.38)
ESRD	**2.74 (1.06**–**7.10)**	**2.96 (1.28**–**6.87)**	**4.05 (1.82**–**8.98)**	1.72 (0.22–13.32)	**3.56 (1.01**–**12.56)**	–	1.40 (0.43–4.61)
Foreign born	**1.77 (1.31**–**2.41)**	0.88 (0.71–1.10)	0.98 (0.68–1.40)	**0.37 (0.19–0.70)**	0.70 (0.41–1.18)	0.97 (0.62–1.51)	0.84 (0.62–1.14)
Previous TB	0.27 (0.07–1.10)	0.80 (0.43–1.49)	1.37 (0.63–2.98)	1.20 (0.37–3.89)	0.37 (0.05–2.70)	0.76 (0.24–2.43)	0.52 (0.20–1.43)

^*a*^*OR* adjusted odds ratio, *CI* confidence interval, *HIV* human immunodeficiency virus, *ESRD* end-stage renal disease

Analysis excluded subjects with multiple subsite involvement (*n* = 57) and laryngeal sites (*n* = 3)

^a^Excessive alcohol use within the past 12 months

^b^HIV unknown categorized as negative

Significant odds ratios (p<0.005) are in bold

### Risk factors for mortality during treatment in patients with exclusively EPTB

Among the 1111 patients with exclusively EPTB who had mortality-related data available, 50 (4.5%) died during anti-TB treatment. Mortality was highest among those patients presenting with meningeal (9.6%) or peritoneal TB (8.5%) and lower among those individuals with lymphatic TB (0.7%) (Fig. 2b). During treatment, no mortality was reported among patients having either laryngeal or multisite TB. Age ≥45 (^a^OR 3.75, 95% CI 1.71–8.22), HIV+ status (^a^OR 4.70, 95% CI 1.54–14.32), excessive alcohol use within the past 12 months (^a^OR 3.34, 95% CI 1.45–7.67), ESRD (^a^OR 4.45, 95% CI 1.38–14.33), and abnormal chest radiographs (^a^OR 2.18, 95% CI 1.09–4.35) were risk factors for TB mortality with adjusted odds ratio (Table 4).

**Table 4:** Risk factors for mortality during anti-tuberculosis treatment in patients with extrapulmonary tuberculosis in Texas, USA, 2009–2015

Variable	Treatment completed (*n* = 1061)	Died during treatment (*n* = 50)	Mortality risk (%)	Crude OR (95% CI)	Adjusted OR (95% CI)	Adjusted *p*-Value
Age (years)						
0–14	105 (9.9%)	0 (0.0%)	0.0%	–		
15–24	116 (10.9%)	2 (4.0%)	1.7%	(reference)		
25–44	438 (41.3%)	9 (18.0%)	2.0%	1.19 (0.25, 5.59)		
45–64	292 (27.5%)	17 (34.0%)	5.5%	3.38 (0.77, 14.85)		
≥65	110 (10.4%)	22 (44.0%)	16.7%	11.60 (2.66, 50.49)		
Age (years)						
<45	659 (62.1%)	11 (22.0%)	1.6%	(reference)	(reference)	
≥45	402 (37.9%)	39 (78.0%)	8.8%	5.81 (2.94, 11.48)	3.75 (1.71, 8.22)	0.001
Gender						
Female	491 (46.3%)	20 (40.0%)	3.9%	(reference)		
Male	570 (53.7%)	30 (60.0%)	5.0%	1.29 (0.72, 2.30)		
Race						
White	84 (7.9%)	9 (18.0%)	9.7%	(reference)		
Black	209 (19.7%)	11 (22.0%)	5.0%	0.49 (0.20, 1.23)		
Hispanic	501 (47.2%)	24 (48.0%)	4.6%	0.45 (0.20, 1.00)		
Asian	262 (24.7%)	6 (12.0%)	2.2%	0.21 (0.07, 0.62)		
Other	5 (0.5%)	0 (0.0%)	0.0%	1.00 (0.00, 0.00)		
Race						
Non-White	977 (92.1%)	41 (82.0%)	4.0%	(reference)		
White	84 (7.9%)	9 (18.0%)	9.7%	2.55 (1.20, 5.43)		
HIV status						
Negative	847 (79.8%)	21 (42.0%)	2.4%	(reference)	(reference)	
Positive	53 (5.0%)	6 (12.0%)	10.2%	4.57 (1.77, 11.79)	4.70 (1.54, 14.32)	0.01
Unknown	161 (15.2%)	23 (46.0%)	12.5%	5.76 (3.11, 10.66)	6.55 (3.19, 13.44)	<0.001
Homeless						
No	1040 (98.0%)	46 (92.0%)	4.2%	(reference)	(reference)	
Yes	21 (2.0%)	4 (8.0%)	16.0%	4.31 (1.42, 13.06)	1.57 (0.40, 6.17)	0.52
Excessive alcohol use						
No	973 (91.7%)	37 (74.0%)	3.7%	(reference)	(reference)	
Yes	88 (8.3%)	13 (26.0%)	12.9%	3.88 (1.99, 7.58)	3.34 (1.45, 7.67)	0.01
Injecting drug use						
No	1050 (99.0%)	48 (96.0%)	4.4%	(reference)		
Yes	11 (1.0%)	2 (4.0%)	15.4%	3.98 (0.86, 18.44)		
Foreign born						
No	415 (39.1%)	29 (58.0%)	6.5%	(reference)		
Yes	646 (60.9%)	21 (42.0%)	3.1%	0.47 (0.26, 0.83)		
Diabetes						
No	948 (89.3%)	40 (80.0%)	4.0%	(reference)	(reference)	
Yes	113 (10.7%)	10 (20.0%)	8.1%	2.10 (1.02, 4.31)	1.55 (0.65, 3.69)	0.32
End-stage renal disease						
No	1043 (98.3%)	44 (88.0%)	4.0%	(reference)	(reference)	
Yes	18 (1.7%)	6 (12.0%)	25.0%	7.90 (2.99, 20.88)	4.45 (1.38, 14.33)	0.01
Immunosuppression (medical condition or medication)						
No	1027 (96.8%)	44 (88.0%)	4.1%			
Yes	34 (3.2%)	6 (12.0%)	15.0%	4.12 (1.64, 10.32)		
Previous TB						
No	1033 (97.4%)	50 (100.0%)	4.6%	–		
Yes	28 (2.6%)	0 (0.0%)	0.0%			
Inmate of a correctional facility						
No	967 (94.8%)	48 (98.0%)	4.7%	(reference)		
Yes	53 (5.2%)	1 (2.0%)	1.9%	0.38 (0.05, 2.81)		
Resident of long-term care facility						
No	1048 (98.8%)	47 (94.0%)	4.3%	(reference)	(reference)	
Yes	13 (1.2%)	3 (6.0%)	18.8%	5.15 (1.42, 18.67)	2.15 (0.50, 9.26)	0.31
AFB smear						
Negative	627 (99.4%)	17 (94.4%)	2.6%	(reference)		
Positive	4 (0.6%)	1 (5.6%)	20.0%	9.22 (0.98, 86.93)		
Culture						
Negative	602 (97.6%)	12 (92.3%)	2.0%	(reference)		
Positive	15 (2.4%)	1 (7.7%)	6.3%	3.34 (0.41, 27.40)		
TB-CXR						
No	611 (62.8%)	15 (33.3%)	2.4%	(reference)	(reference)	
Yes	362 (37.2%)	30 (66.7%)	7.7%	3.38 (1.79, 6.36)	2.18 (1.09, 4.35)	0.03
Cavitation on CXR						
No	354 (97.8%)	30 (100.0%)	7.8%	–		
Yes	8 (2.2%)	0 (0.0%)	0.0%			
DST profile						
Sensitive to RIF and INH	560 (52.8%)	40 (80.0%)	6.7%			
Resistant to RIF or INH	39 (3.7%)	0 (0.0%)	0.0%			
MDR-TB	3 (0.3%)	0 (0.0%)	0.0%	(reference)		
Unavailable	459 (43.3%)	10 (20.0%)	2.1%	0.31 (0.15, 0.62)		
Genotyping lineage						
Indo-Oceanic (L1)	80 (7.5%)	4 (8.0%)	4.8%	0.79 (0.20, 3.05)		
East Asian (L2)	79 (7.4%)	5 (10.0%)	6.0%	(reference)		
East African-Indian (L3)	35 (3.3%)	1 (2.0%)	2.8%	0.45 (0.05, 4.01)		
Euro-American (L4)	300 (28.3%)	27 (54.0%)	8.3%	1.42 (0.53, 3.81)		
* M. bovis*	27 (2.5%)	3 (6.0%)	10.0%	1.76 (0.39, 7.84)		
Other	8 (0.8%)	0 (0.0%)	0.0%	1.00 (0.00, 0.00)		
Unknown	532 (50.1%)	10 (20.0%)	1.8%	0.30 (0.10, 0.89)		
Genotyping lineage						
Not East Asian	354 (97.8%)	30 (100.0%)	7.8%	(reference)	(reference)	
East Asian (L2)	8 (2.2%)	0 (0.0%)	0.0%	1.38 (0.53, 3.58)	1.58 (0.53, 4.74)	0.42

*TB* tuberculosis, *HIV* human immunodeficiency virus, *TB-CXR* tuberculosis chest X-ray, *DST* drug susceptibility test, *RIF* rifampin, *INH* isoniazid, *MDR-TB* multidrug-resistant tuberculosis, *OR* odds ratios

Analysis was performed on 1111 patients with extrapulmonary tuberculosis only and having treatment outcome information available

## Discussion

Although risk factors for the development of exclusively EPTB compared to PTB have been described in several studies^5, 12, 16, 17^, there are still inconsistent findings among studies from different regions, including substantial state-level heterogeneity in the reported epidemiological data^18^. We performed an analysis of EPTB patients in the state of Texas. We found that patients who were age ≥45 years, female, HIV+, and suffering from ESRD were at a significantly elevated risk of EPTB. In particular, age ≥45 years, HIV+, excessive alcohol use within the past 12 months, ESRD, and abnormal chest radiographs were risk factors for EPTB mortality during treatment.

The observed demographics of female gender and foreign-born origin in the USA have been previously reported as risk factors for EPTB^5, 12, 17, 19, 20^. Similarly, we found that female gender and Hispanic ethnicity were associated with patients who presented with exclusively EPTB after adjusting for other confounding factors. We further noted that ethnicity was not associated with any specific site of EPTB and that female gender was associated with lymphatic and peritoneal TB.

The risk of TB development in the foreign-born population was substantially elevated even more than 5 years after entering the USA^20^. In our study population, more than half (55.2%) of the patients were born outside the USA. The cervical lymphatic site was found to be a more common disease site among foreign-born EPTB patients than among US-born patients in North Carolina^21^. However, a study at a single urban US public hospital did not show any risk between foreign birth with sites of EPTB^13^. From our results, foreign birth was associated with increased risk of pleural TB. Pleural TB is the second most common type of EPTB but the leading manifestation in settings with high TB^22, 23^. Given that TB disease among foreign-born populations is normally considered to result from the reactivation of LTBI^24^, one explanation might be that pleural TB was the primary manifestation of LTBI reactivation^22^. It is also possible that the association between foreign birth and pleural TB is due to the effect of BCG vaccination on foreign-born individuals. Studies have shown that pleuritis is induced by *Mycobacterium bovis* BCG, and the underlying mechanisms have been elucidated^25^. However, non-association of BCG status among patients with PTB, pleural TB, and other types of EPTB was seen in a study with a majority of BCG-vaccinated patients^23^. From the findings described herein, studies can be initiated to further evaluate the BCG status of foreign-born patients in relation to pleural TB.

Drug users and the elderly remain the groups at high risk of developing LTBI and TB disease^19^. The prevalence of illicit drug use has been previously shown to be higher in patients with PTB than in patients with EPTB^15, 17^. Similarly, the prevalence of injecting drug use and non-injecting drug use was also higher in patients with PTB than patients with exclusively EPTB in our study. We further observed that injecting drug use was associated with patients with genitourinary TB. In addition, non-injecting drug use as a non-risk factor for EPTB compared to PTB has been reported^26^. From our results, non-injecting drug use was also negatively associated with patients having exclusively EPTB or any EPTB compared to PTB. We demonstrated that the age groups 15–24 and 45–64 were associated with exclusively EPTB. Meanwhile, Click et al.^12^ indicated that age 0–4 was associated with exclusively EPTB. TB in elderly patients can involve almost any organ in the body^27^. We found that patients age >45 years were at an increased risk of bone TB but not with other extrapulmonary sites. This finding is consistent with a large-population-based study in Europe^16^. Additionally, ages <15 and >65 years were likely to be associated with the most comment types of EPTB^15^.

According to a 2016 CDC report, high rates of TB cases overall and TB cases attributable to extensive recent transmission were identified frequently among people experiencing homelessness within the past year and residents of a correctional facility at the time of diagnosis^28^. However, EPTB was more common in non-homeless patients than in the homeless, as previously reported^5, 17^. The number of homeless cases was significantly higher in PTB (5.8%) than in exclusively EPTB (2.4%) in our population. Furthermore, we showed that being homeless was negatively associated with EPTB and especially lymphatic TB. Neither the Magee study nor our study showed any significant differences in the proportion of residents of correctional facility patients with exclusively EPTB vs. PTB^17^. These findings suggest that *Mtb* infection is not site-specific in correctional facility patients.

It is well known that patients with HIV have an increased risk of EPTB^13, 14^. Click et al.^12^ found that HIV was associated with both exclusively EPTB and any EPTB. Moreover, the same study demonstrated that the association between individuals with HIV infection and extrapulmonary disease was greater for EPTB with concurrent pulmonary involvement than for exclusively EPTB alone. In our population, HIV was associated with EPTB with concurrent pulmonary involvement and any EPTB but not with exclusively EPTB. We further identified that HIV was associated with meningeal TB at more than five times the odds, while other sites were not found to be related. In addition, low CD4 lymphocyte counts in hospitalized patients with HIV-co-infected EPTB were found to be associated with meningeal TB and disseminated TB in a single hospital study in the USA^13^.

The prevalence of end-stage renal disease (ESRD) continues to rise by approximately 20,000 cases per year in the USA^29^. Consistent epidemiologic evidence has shown that this population is at risk for developing active TB^30^. ESRD as a risk factor for EPTB has also been reported in studies in Taiwan and the US state of Georgia^17, 31^. Accordingly, ESRD was associated with exclusively EPTB, EPTB with concurrent pulmonary involvement, and all cases of EPTB compared to PTB in our population-based analysis. We also found that ESRD was specifically associated with pleural, lymphatic, bone and peritoneal TB. Thus, patients with ESRD are at a high risk of progressing to EPTB. One possibility regarding the underlying mechanisms is that the persistence of impaired cell-mediated immunity in ESRD may leave these patients susceptible to *Mtb* infection or activation of latent infection^32^. This phenomenon has also been observed in organ transplantation receipts who receive post-transplant immunosuppressive medications that specifically target T cell-mediated immunity^33, 34^. In regard to the screening strategy among patients with chronic renal disease (CRD), both the American Thoracic Society^35^ and American Transplant Society^36^ guidelines recommend that all immunocompromised subjects and transplant candidates be screened for TB with a tuberculin skin test (TST) or IFN-γ releasing assay (IGRA). The WHO provides more specific guidance on screening all dialysis patients with TST or IGRAs^37^. Beyond the screening strategy, diagnosis of EPTB remains difficult because of the paucibacillary nature. Thus, it is necessary to advance knowledge about the association of CRD/ESRD and EPTB and strategies to improve the prevention, detection, and treatment of EPTB with ESRD.

Consistent with the current literature, our analyses showed that age ≥45 years, ESRD, HIV+ status, excessive alcohol use within the past 12 months, and abnormal chest radiography were significantly associated with mortality during anti-TB treatment in patients with exclusively EPTB^17, 38^. In the USA, excess alcohol use may represent a large portion of TB burden^38^. The relationship between excessive alcohol use and the development of TB disease as well as TB-associated morbidity and mortality has been presumed to be due to impaired immune function^39^. Other confounding factors such as older age, diabetes, ESRD, and HIV are all related to decreased immune function. HIV was highly associated with mortality during treatment in our analysis, which is consistent with other epidemiologic and observational studies with mortality ranging from 6% to 32%^40^. Another important finding of our study was that ESRD was associated with more than quadruple the odds of mortality during anti-TB treatment in patients having exclusively EPTB. Given these findings, we suggest that patients with decreased immune function or immunosuppression are at an increased risk of mortality during treatment and that host immune response may determine the difference in survival. Approaches to meet the objective of optimizing efficacy and safety of treatment, especially for TB-HIV co-infection as well as ESRD, to reduce mortality both in adults and children are urgently required. As shown in a prospective cohort study that enrolled hospitalized HIV co-infected patients with microbiologically confirmed drug-susceptible TB in South Africa, mortality within 12 weeks was positively associated with elevated concentrations of procalcitonin, activation of the innate immune system, and anti-inflammatory markers^41^. Procalcitonin, a product induced by TNF-α and IL-2 during a bacterial infection, has already shown its value in distinguishing TB from bacterial pneumonia and TB meningitis from bacterial meningitis^42, 43^. A higher level of serum procalcitonin was associated with a poorer prognosis in TB meningitis^43^. Moreover, serum procalcitonin has been reported as an appropriate indicator of infection in ESRD patients^44^. Therefore, identifying a correlation between the host’s immunologic phenotypes and the severity of disease or comorbidities, as well as treatment response, would enable the direct selection of host-directed therapeutics and a potentially beneficial and improved TB outcome.

Another important consideration for the risk of EPTB is diabetes status, as several studies have identified diabetes as a risk factor for developing active TB and poor treatment outcomes^45^. For instance, a study in the UK has reported that patients with diabetes had an increased risk of developing TB compared to a control group^46^. Among patients undergoing TB treatment, patients with diabetes had an increased mortality risk compared to those without diabetes^47^. However, compared to the control group, TB patients with diabetes had an increased probability of having PTB as opposed to EPTB^46^. Furthermore, both our study and a cohort study in the US state of Georgia found TB patients with diabetes to have an increased probability of EPTB as opposed to PTB, and diabetes was not associated with EPTB mortality during TB treatment^17^. In an additional study, diabetes did not contribute to TB-related death in adult patients in the USA^48^. Thus, the inconsistent findings encourage further investigations on the impact of diabetes in PTB and EPTB patients. It is worth emphasizing that the incidence of ESRD is higher in the diabetic population than in the non-diabetic population^49^. Since ESRD is an independent risk factor for EPTB as shown in our study, it may be necessary to account for the association between diabetes and ESRD as a co-epidemic, which would potentially account for the risk of EPTB as well as treatment outcomes.

Among available data for patients with exclusively EPTB, the most prevalent *Mtb* lineages were Euro-American L4, followed by East Asian L2 and Indo-Oceanic L1. This finding was consistent with a previous nationwide study in the USA^12^. Click et al.^12^ suggested that the percentage of cases with exclusively EPTB differs for the four lineages—East Asian, 13.0%; Euro-American, 13.8%; Indo-Oceanic, 22.6%; and East African-Indian, 34.3%—while EPTB with pulmonary involvement did not. However, Click’s study did not show data for *M. bovis*. Consistent with the results of previous studies^50, 51^, we found a higher proportion of *M. bovis* in patients with exclusively EPTB than in patients with exclusively PTB or EPTB with pulmonary involvement. Geographically, patients with *M. bovis* TB residing along the US–Mexico border had a disproportionately high incidence of *M. bovis*^50^. This may also be reflected in our results, as Texas is a US state bordering Mexico. The tradition of raw milk and cheese consumption, especially in Hispanic communities, may be another common reason for *M. bovis* infection^52^. Additionally, we found that positive smear and culture, direct susceptibility test profile and genotyping lineage were not risk factors for mortality from exclusively EPTB during TB treatment. These results may be limited due to many individuals with unknown *Mtb* status, either because the test was not performed or because the results were not recorded. Instead of bacterial factors, host risk factors such as HIV, age ≥45, excessive alcohol use, and abnormal chest X-ray findings were associated with mortality during treatment of EPTB in our study.

One important limitation of our study is the unavailability of data in some categories. Therefore, the reported crude and adjusted odds ratios could be biased due to unmeasured covariates or unknown confounders. State TB surveillance reporting does not include the depth of clinical information necessary to further investigate recognized risk factors in the epidemiology of EPTB (e.g., CD4 lymphocyte counts for HIV patients and smoking status). An observational design in a large cohort will be necessary to assess the true effect of these factors. Given that Texas has one of the highest TB prevalence rates of any US state and has a more diverse population than many other states, findings from our analysis may not apply to settings elsewhere in the country.

## Conclusion

The present study characterized the important differences in the population-level dynamics of EPTB, as well as its specific sites, which included demographic factors and clinical characteristics in addition to the heterogeneities within sites of EPTB during the 7-year study period. Age ≥45 years, HIV+ status, and ESRD were identified as risk factors for both EPTB establishment and resulting mortality during treatment. Although the scientific question of the extrapulmonary dissemination remains to be answered, the study’s findings could allow us to design supportive treatments for specific subgroups of patients with increased mortality, such as those with a HIV+ status and those with compromised renal function, in order to improve their outcomes and ultimately minimize the transmission of TB.

## References

[1] World Health Organization. *Global Tuberculosis Report* (2016). http://www.who.int/tb/publications/global_report/en/

[2] Pai M (2016). Tuberculosis. Nat. Rev. Dis. Primers.

[3] Sandgren A, Hollo V, van der Werf MJ (2013). Extrapulmonary tuberculosis in the European Union and European Economic Area, 2002 to 2011. Eur. Surveill..

[4] Sama JN (2016). High proportion of extrapulmonary tuberculosis in a low prevalence setting: a retrospective cohort study. Public Health.

[5] Peto HM, Pratt RH, Harrington TA, LoBue PA, Armstrong LR (2009). Epidemiology of extrapulmonary tuberculosis in the United States, 1993-2006. Clin. Infect. Dis..

[6] Centers for Disease Control and Prevention. Reported Tuberculosis in the United States 2013. http://www.cdc.gov/features/dstuberculosis

[7] Lin PL (2014). Sterilization of granulomas is common in active and latent tuberculosis despite within-host variability in bacterial killing. Nat. Med..

[8] Via LE (2015). Host-mediated bioactivation of pyrazinamide: implications for efficacy, resistance, and therapeutic alternatives. ACS Infect. Dis..

[9] Martin CJ (2017). Digitally barcoding Mycobacterium tuberculosis reveals in vivo infection dynamics in the Macaque model of tuberculosis. mBio.

[10] Lieberman TD (2016). Genomic diversity in autopsy samples reveals within-host dissemination of HIV-associated Mycobacterium tuberculosis. Nat. Med..

[11] Whittaker E, Nicol M, Zar HJ, Kampmann B (2017). Regulatory T cells and pro-inflammatory responses predominate in children with tuberculosis. Front. Immunol..

[12] Click ES, Moonan PK, Winston CA, Cowan LS, Oeltmann JE (2012). Relationship between Mycobacterium tuberculosis phylogenetic lineage and clinical site of tuberculosis. Clin. Infect. Dis..

[13] Leeds IL (2012). Site of extrapulmonary tuberculosis is associated with HIV infection. Clin. Infect. Dis..

[14] Sterling TR (2001). Human immunodeficiency virus-seronegative adults with extrapulmonary tuberculosis have abnormal innate immune responses. Clin. Infect. Dis..

[15] Gonzalez OY (2003). Extra-pulmonary manifestations in a large metropolitan area with a low incidence of tuberculosis. Int. J. Tuberc. Lung Dis..

[16] Sotgiu G (2017). Determinants of site of tuberculosis disease: an analysis of European surveillance data from 2003 to 2014. PLoS ONE.

[17] Magee MJ, Foote M, Ray SM, Gandhi NR, Kempker RR (2016). Diabetes mellitus and extrapulmonary tuberculosis: site distribution and risk of mortality. Epidemiol. Infect..

[18] Shrestha S, Hill AN, Marks SM, Dowdy DW (2017). Comparing drivers and dynamics of tuberculosis in California, Florida, New York, and Texas. Am. J. Respir. Crit. Care. Med..

[19] Deiss RG, Rodwell TC, Garfein RS (2009). Tuberculosis and illicit drug use: review and update. Clin. Infect. Dis..

[20] Cain KP (2007). Tuberculosis among foreign-born persons in the United States: achieving tuberculosis elimination. Am. J. Respir. Crit. Care. Med..

[21] Kipp AM, Stout JE, Hamilton CD, Van Rie A (2008). Extrapulmonary tuberculosis, human immunodeficiency virus, and foreign birth in North Carolina, 1993-2006. BMC Public Health.

[22] Jeon D (2014). Tuberculous pleurisy: an update. Tuberc. Respir. Dis. (Seoul).

[23] Rasolofo Razanamparany V, Menard D, Auregan G, Gicquel B, Chanteau S (2002). Extrapulmonary and pulmonary tuberculosis in Antananarivo (Madagascar): high clustering rate in female patients. J. Clin. Microbiol..

[24] Tsang CA, Langer AJ, Navin TR, Armstrong LR (2017). Tuberculosis among foreign-born persons diagnosed /=10 years after arrival in the United States, 2010-2015. Am. J. Transplant..

[25] Chavez-Galan L (2017). Transmembrane tumor necrosis factor controls myeloid-derived suppressor cell activity via TNF receptor 2 and protects from excessive inflammation during BCG-induced pleurisy. Front. Immunol..

[26] Yang Z (2004). Identification of risk factors for extrapulmonary tuberculosis. Clin. Infect. Dis..

[27] Rajagopalan S (2016). Tuberculosis in older adults. Clin. Geriatr. Med..

[28] Centers for Disease Control and Prevention. Reported Tuberculosis in the United States 2016. http://www.cdc.gov/features/dstuberculosis.

[29] United States Renal Data System. *USRDS 2017 Annual Data Report: Atlas of Chronic Kidney Disease and End-Stage Renal Disease in the United States* (National Institutes of Health, National Institute of Diabetes and Digestive and Kidney Diseases, Bethesda, MD). https://www.usrds.org/2017/view/v2_01.aspx.

[30] Al-Efraij K (2015). Risk of active tuberculosis in chronic kidney disease: a systematic review and meta-analysis. Int. J. Tuberc. Lung Dis..

[31] Lin JN (2009). Risk factors for extra-pulmonary tuberculosis compared to pulmonary tuberculosis. Int. J. Tuberc. Lung Dis..

[32] Kato S (2008). Aspects of immune dysfunction in end-stage renal disease. Clin. J. Am. Soc. Nephrol..

[33] Mysore KR (2017). Longitudinal assessment of T cell inhibitory receptors in liver transplant recipients and their association with posttransplant infections. Am. J. Transplant..

[34] Liyanage T (2015). Worldwide access to treatment for end-stage kidney disease: a systematic review. Lancet.

[35] Lewinsohn DM (2017). Official American Thoracic Society/Infectious Diseases Society of America/Centers for Disease Control and Prevention Clinical Practice Guidelines: diagnosis of tuberculosis in adults and children. Clin. Infect. Dis..

[36] Morris MI (2012). Diagnosis and management of tuberculosis in transplant donors: a donor-derived infections consensus conference report. Am. J. Transplant..

[37] WHO. *Guidelines on the Management of Latent Tuberculosis Infection* (World Health Organization, Geneva, Switzerland, 2015). 25973515

[38] Volkmann T, Moonan PK, Miramontes R, Oeltmann JE (2015). Tuberculosis and excess alcohol use in the United States, 1997-2012. Int. J. Tuberc. Lung Dis..

[39] Happel KI, Nelson S (2005). Alcohol, immunosuppression, and the lung. Proc. Am. Thorac. Soc..

[40] Odone A (2014). The impact of antiretroviral therapy on mortality in HIV positive people during tuberculosis treatment: a systematic review and meta-analysis. PLoS ONE.

[41] Janssen S (2017). Mortality in severe human immunodeficiency virus-tuberculosis associates with innate immune activation and dysfunction of monocytes. Clin. Infect. Dis..

[42] Huang SL (2014). Value of procalcitonin in differentiating pulmonary tuberculosis from other pulmonary infections: a meta-analysis. Int. J. Tuberc. Lung Dis..

[43] Kim J (2016). Procalcitonin as a diagnostic and prognostic factor for tuberculosis meningitis. J. Clin. Neurol..

[44] Lee WS (2015). Cutoff value of serum procalcitonin as a diagnostic biomarker of infection in end-stage renal disease patients. Korean J. Intern. Med..

[45] Critchley JA (2017). Defining a research agenda to address the converging epidemics of tuberculosis and diabetes: Part 1: Epidemiology and clinical management. Chest.

[46] Young F, Wotton CJ, Critchley JA, Unwin NC, Goldacre MJ (2012). Increased risk of tuberculosis disease in people with diabetes mellitus: record-linkage study in a UK population. J. Epidemiol. Community Health.

[47] Degner NR, Wang JY, Golub JE, Karakousis PC (2018). Metformin use reverses the increased mortality associated with diabetes mellitus during tuberculosis treatment. Clin. Infect. Dis..

[48] Beavers, S. F., et al. Tuberculosis mortality in the United States: epidemiology and prevention opportunities. *Ann. Am. Thorac. Soc*. (2018) [Epub ahead of print]. 10.1513/AnnalsATS.201705-405OC PMC6531349 29490150

[49] Narres M (2016). The incidence of end-stage renal disease in the diabetic (compared to the non-diabetic) population: a systematic review. PLoS ONE.

[50] Scott C (2016). Human tuberculosis caused by Mycobacterium bovis in the United States, 2006-2013. Clin. Infect. Dis..

[51] Majoor CJ, Magis-Escurra C, van Ingen J, Boeree MJ, van Soolingen D (2011). Epidemiology of Mycobacterium bovis disease in humans, The Netherlands, 1993-2007. Emerg. Infect. Dis..

[52] Hlavsa MC (2008). Human tuberculosis due to Mycobacterium bovis in the United States, 1995-2005. Clin. Infect. Dis..

